# Sleep disturbance as a transdiagnostic marker of children's mental health difficulties: A network analysis of item‐level associations between different types of sleep problems and different behavioural and emotional symptoms

**DOI:** 10.1002/jcv2.70104

**Published:** 2026-03-04

**Authors:** Alina A. Marinca, Julia E. Michalek, Alice M. Gregory, Afia Ali, Jennifer Y. F. Lau

**Affiliations:** ^1^ Centre for Psychiatry and Mental Health Wolfson Institute of Population Health Youth Resilience Unit Queen Mary University of London London UK; ^2^ Department of Psychology Royal Holloway University of London London UK

**Keywords:** behavioural symptoms, children, emotional, network, sleep, transdiagnostic

## Abstract

**Background:**

Sleep disturbances are widely considered to be a transdiagnostic feature of common behavioural and emotional difficulties in childhood, yet most studies treat sleep as a single construct. Where studies have explored specific sleep problems to psychopathology in children, these tend to only include behavioural or emotional difficulties, ignoring the often‐observed covariance between them. These study limitations kerb our knowledge over whether individual sleep disturbances are universal (transdiagnostic) or specific to individual symptom types.

**Objectives:**

To address these gaps, we aimed to map item‐level associations between specific sleep problems and behavioural and emotional symptoms using a network approach. We predicted that nodes would organise into clusters corresponding to behavioural symptoms, emotional symptoms, and various sub‐types sleep problems. We also expected sleep problems to be central in the network.

**Methods:**

We used data from the Development of Emotional Resilience cohort, which comprises a sample of children living in East London (*N* = 876, aged 8–12 years; 47.7% male; 24.6% in free school meals; 57.5% British Asian) and applied network analysis to examine associations between self‐reported sleep problems, teacher‐reported behavioural symptoms (hyperactivity/impulsivity, inattention, conduct) and self‐reported emotional symptoms (anxiety, depression).

**Results:**

Seven symptom clusters emerged, reflecting distinct clusters of behavioural symptoms, some mixed sleep‐emotional symptoms, and mixed sleep problems. Node centrality analyses identified anxiety and sleep anxiety items as the most central nodes in the network. Among the top bridge nodes linking clusters in the network were problems with appetite, trouble sleeping, daytime sleepiness, and anticipatory worry about bedtime, potentially reflecting transdiagnostic pathways across symptom domains.

**Conclusion:**

Sleep and bedtime‐related anxiety may act as central, transdiagnostic mechanisms, proximally in relation to emotional symptoms and distally in relation to behavioural symptoms. Targeted interventions focussing on sleep anxiety may benefit children across symptom domains.

## Key Points

**What's known?**

- Sleep disturbances are widely considered a transdiagnostic feature of childhood emotional and behavioural difficulties, yet most studies treat sleep as a single construct.

**What's new?**

- Network analysis in an under‐represented socio‐economically and culturally‐diverse aged‐school children cohort revealed seven clusters for emotional symptoms, behavioural symptoms, and sleep problems. Node centrality analyses identified sleep anxiety items among the most central nodes, followed by depressive symptoms. Behavioural symptoms were more peripheral.

**What's relevant?**

- Sleep problems act as central, transdiagnostic mechanisms, particularly for emotional symptoms. Targeted interventions addressing sleep anxiety and emotional regulation may benefit multiple symptom domains and inform clinical practice, policy, and early intervention programs.

## INTRODUCTION

Behavioural symptoms such as hyperactivity, inattention, and aggressive or antisocial behaviour (sometimes referred to as sources of neurodivergence) and emotional symptoms, such as anxiety and depression, have risen dramatically in children following the COVID‐19 pandemic (Yıldırım Budak et al., 2024). Untreated early‐onset mental health difficulties can persist and pose considerable healthcare costs globally (World Health Organization, 2021). Strikingly, these symptom types often co‐occur from early school years (Walker et al., 2025; Willner et al., 2016). Prevalence estimates for comorbid behavioural and emotional symptoms in children vary considerably, ranging from 2.4% to 34.4% across different study populations and contexts (Danielson et al., 2021; Nivard et al., 2017). Children with comorbid symptoms consistently experience poorer academic, social, and mental health outcomes compared to those exhibiting only one symptom type (Papachristou & Flouri, 2020; Shi & Ettekal, 2021). Searching for shared underlying processes (‘transdiagnostic mechanisms’) may assist in early identification of mental health difficulties and inform the development of universal interventions. Sleep problems are regarded a transdiagnostic risk factor for many mental health disorders including externalising and internalising conditions (Harvey & Sarfan, 2024). However, as sleep problems themselves comprise a wide‐ranging set of difficulties relating to sleep behaviours, routines, emotions and quality, it remains unclear which sleep problems associate with both symptom types. Here, we map the more nuanced associations between sleep problems and symptom‐types with network analysis.

Sleep problems affect 25%–50% of children globally and are more recognised since the pandemic (Bruni et al., 2025; Tamir et al., 2023). They are a part of the diagnostic criteria for many mental illnesses (e.g., depression, anxiety, bipolar disorder; American Psychiatric Association (2013), DSM‐5) and predict the onset of many of these conditions (Harvey et al., 2021; Kivelä et al., 2018; Sarfan et al., 2023) including childhood behavioural and emotional problems, and their comorbidity (Bruni et al., 2025; Sesso et al., 2024). Yet, ‘sleep problems’ encompass a heterogeneous set of difficulties (Harvey & Sarfan, 2024), manifesting as challenges around sleep onset, duration, and quality, as well as difficulties falling and staying asleep. Sleep problems can also include (a) parasomnias, unusual or disruptive behaviours or experiences that occur during the transition into sleep, within sleep, or during sleep (Becker et al., 2020; Bruni et al., 2025), (b) nightmares, night terrors, restless sleep and certain forms of night waking (one or more awakenings after initially falling asleep), (c) challenging bedtime routines such as bedtime resistance and sleep anxiety that is, fears around sleep or the dark that may result in a reluctance to sleep alone, and (d) the consequences of poor sleep including daytime sleepiness and worse daytime functioning.

While historically studies tended to measure composite sleep problem scores, limiting knowledge over which specific sleep problems relate to specific behavioural and emotional symptoms, some have explored more differentiated associations between different aspects of sleep and psychopathology. Shorter sleep duration longitudinally predicts hyperactivity and inattention symptoms (Touchette et al., 2007). Shorter sleep duration also characterizes children with conduct problems (Sidol et al., 2023; Tomasiello et al., 2021) and anxiety and depression symptoms (Hamilton et al., 2007; Scarpelli et al., 2021). Parasomnias have been found in children with ADHD and conduct problems, accompanying other sleep issues such as shorter sleep duration, bedtime resistance, and night wakings (Smedje et al., 2001). Parasomnias are also observed in children with anxiety and depression (Becker et al., 2020; Dong et al., 2024). Bedtime resistance and sleep anxiety have been associated with hyperactivity, inattention, conduct problems, and emotional symptoms (Dong et al., 2024; Fletcher et al., 2018; Wagner & Schlarb, 2012) as does daytime sleepiness due to poor sleep quality, which can exacerbate core symptoms and mood dysregulation (Dong et al., 2024; Touchette et al., 2007; Wagner & Schlarb, 2012).

Taken together, it may be tempting to conclude that specific sleep problems are universally associated with all symptom types. However, most of these report on ‘univariate’ associations, that is, where a specific sleep problem is associated with either emotional or behavioural symptoms without statistically controlling for the other. Thus, associations may be confounded by unmeasured covariance between behavioural and emotional symptoms, limiting our ability to generate precise predictions on differential associations. In network analysis (Hevey, 2018), we can explore more item‐level associations between specific sleep problems and specific symptom‐types from within emotional (anxiety, depression) and behavioural (hyperactivity/impulsivity, inattention, conduct) domains while controlling for shared variance between emotional and behavioural symptoms. Network models represent interconnections (edges) between individual variables (nodes) and can illustrate how these variables influence, depend on, or relate to one another. Within a network, models identify clusters of items that aggregate together. They also highlight central items, which have the strongest and most numerous direct connections, as well as bridging items that link separate clusters and facilitate the spread of influence across communities. A network model thus offers a powerful framework to decompose and understand more complex, multifactorial phenomena (Hevey et al., 2018).

We used a network model approach here. While other recent studies have also applied network analysis to sleep and child psychopathology (Richdale et al., 2024; Sommers et al., 2025), these have been conducted in clinical samples with autism. Although insightful, these findings may not generalise to non‐clinical samples where these relationships may differ. Here we extend this work by examining associations between individual sleep problems and behavioural and emotional symptoms in an selected sample of school‐aged children. We also extend previous network analysis work exploring item‐level associations, thus taking a deeper look into specific sleep problems and specific symptoms, rather than sub‐scale scores that collapse across items (Richdale et al., 2024; Sommers et al., 2025). We had two predictions. First, within the network, we expected to find distinct clusters that reflected the different types of behavioural problems (e.g., ADHD symptoms of hyperactivity and inattention items, conduct problems), different types of emotional problems (e.g., anxiety, depression) and different types of sleep problems as reflected in the wider literature (e.g., bedtime resistance, sleep onset, sleep duration, sleep anxiety, waking in the night, parasomnias, daytime sleepiness; Owens et al., 2000; Fulfs et al., 2024) that were being captured in the study. Second, we expected sleep problems to be central to the network given their widespread connections with other symptom clusters. However, as prior ‘univariate’ data shows that all sleep problems link with all symptom‐types, we did not have specific predictions on which sleep problems are linked with which symptom‐types. In exploratory analysis we examined whether the specific associations and strength of associations between sleep problems and mental health symptoms differ between boys and girls. This was of interest given clear male‐female differences when considering behavioural and emotional symptoms independently (Mendolia et al., 2021; Njardvik et al., 2025), even though these sex differences are less consistent in the co‐occurrence of symptoms (Diamantopoulou et al., 2011; Poirier et al., 2015) and in their associations with sleep problems.

## METHODS

### Sample

The Development of Emotional Resilience observational cohort (van Loggerenberg et al., 2025) is a 3‐year longitudinal study investigating risk and resilience factors in children attending 10 primary schools in East London. East London comprises boroughs such as Newham, Redbridge, Waltham Forest, and Havering, some areas of which there are high levels of child poverty (Londonmapper, 2023; On London, 2023). Children with a diagnosis of Intellectual Disability Disorder or perceived not to understand the assessments were excluded. This exclusion was applied because the study relied on child self‐report measures that may not be validated or suitable for children with intellectual disabilities (van Loggerenberg et al., 2025). Ethical approval was obtained from the Queen Mary Research Ethics Committee (QMREC) (ref: QMERC22.251). Participants were recruited from 10 schools across East London using a combination of opt‐in and opt‐out consent procedures. Parental consent and child assent were obtained before participation. All planned analyses were preregistered on the Open Science Framework (OSF) (https://osf.io/s5ypg). Baseline data collection (T1) took place between November 2022 and November 2023 but as sleep was not assessed at T1, for this pre‐registered analysis, we used data collected at wave 2 (T2), collected between February 2024 to October 2024. In small groups, across three testing sessions, children used the Redcap mobile application on Android tablets (supported by research assistants) to report on their sleep problems and emotional symptoms. Child behavioural symptoms were reported by their class teachers and all demographic information was provided by the schools.

The final T2 sample comprises 876 participants aged 8–12 years. Just under half (47.7%) are male and many participants are British Asian (57.5%). About a quarter (24.6%) receive free school meals (a marker of social deprivation/lower socio‐economic status, Hobbs & Vignoles, 2010). The age range for our study was 8–12 years old with a mean age of 9.90 (SD = 0.88).

### Measures

#### Sleep problems

**FIGURE 1:**
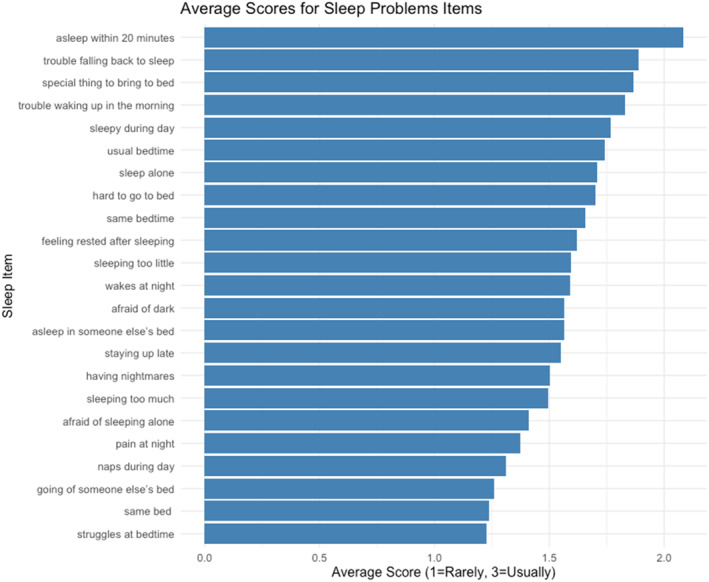
Average scores for individual sleep problem items from the child‐reported SSR. The *X* axis represents the average score for each sleep problems item, with possible scores ranging from 1 (Rarely) to 3 (Usually). Higher values indicate more frequent occurrence of the sleep problem. The *Y* axis lists the individual sleep problems items from the child‐reported SSR.

These were measured by Sleep Self‐report (SSR; Owens et al., 2000). The SSR is a 26‐item scale measuring sleep habits and common sleep disturbances in school aged children. Twenty‐three items of the scale (Figure 1) measure a diverse range of sleep problems on a 3‐point Likert scale: ‘rarely’ (0–1/week or never), ‘sometimes’ (2–4 times/week) or ‘usually’ (5–7/week). With some positively‐phrased items reverse coded, higher scores consistently reflected more sleep problems. Additionally, three items asked if children liked going to sleep, if they had trouble sleeping, and who set rules around bedtime (Owens et al., 2000). The SSR correlates with parent‐report sleep measures (Meltzer et al., 2013; Owens et al., 1998) and showed good reliability in the present sample (*α* = 0.79).

#### Behavioural symptoms

**FIGURE 2:**
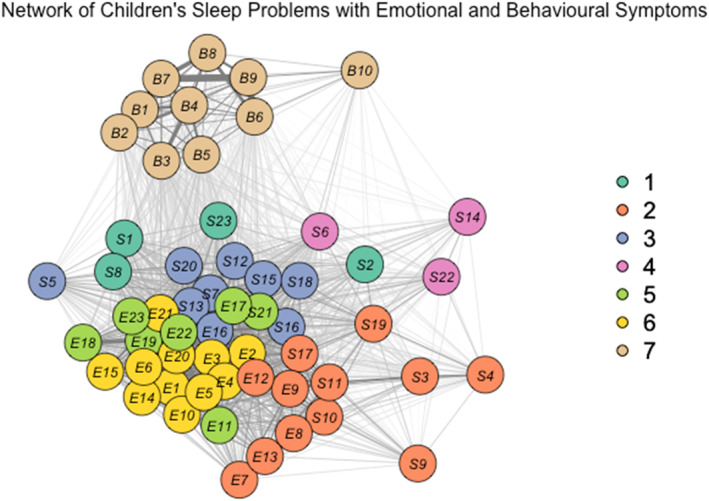
The main network model. Network of children's sleep problems, emotional, and behavioural symptoms. Nodes represent variables: S1–S23 = sleep problems variables, E1–E23 = emotional symptoms variables, B1–B10 = behavioural symptoms variables. Node colours indicate Louvain community clusters. Edges are weighted by correlation strength. Network estimated using RStudio (Version 2024.12.0 + 467), packages: qgraph 1.9.12, igraph 1.4.0. Full item descriptions for all nodes included in the network. *Sleep problems (SSR)*: S1 = Do you go to bed at the same time every night on school nights? (reverse codded); S2, = Do you fall asleep in the same bed every night? (reverse coded); S3 = Do you fall asleep alone? (reverse coded); S4, = Do you fall asleep in parents', brothers', or sisters' bed?, S5 = Do you fall asleep in about 20 min? (reverse coded), S6 = Do you fight with your parents about going to bed?; S7 = Is it hard for you to go to bed?; S8 = Are you ready for bed at your usual bedtime? (reverse coded); S9 = Do you have a special thing (doll, blanket, etc.) you bring to bed?; S10 = Are you afraid of the dark?; S11 = Are you afraid of sleeping alone?; S12 = Do you stay up late when your parents think you are asleep?; S13 = Do you think you sleep too little?,; S14 = Do you think you sleep too much?; S15 = Do you wake up at night when your parents think you're asleep?; S16 = Do you have trouble falling back to sleep if you wake up during the night?; S17 = Do you have nightmares?; S18 = Does pain wake you up at night?; S19 = Do you sometimes go to someone's bed during the night?; S20 = Do you have trouble waking up in the morning?; S21 = Do you feel sleepy during the day?; S22 = Do you take naps during the day?; S23 = Do you feel rested after a night's sleep? (reverse coded). *Emotional symptoms (RCADS)*: E1 = I worry about things; E2 = I worry that something awful will happen to someone in my family; E3 = I worry that bad things will happen to me; E4 = I worry that something bad will happen to me; E5 = I worry about what is going to happen; E6 = I think about death; E7 = I would feel afraid of being on my own at home; E8 = I worry about being away from my parents; E9 = I feel scared if I have to sleep on my own; E10 = I have trouble going to school in the mornings because I feel nervous or afraid; E11 = I am afraid of being in crowded places (like shopping centres, the movies, buses, busy playgrounds); E12 = I worry when I go to bed at night; E13 = I would feel scared if I had to stay away from home overnight; E14 = I feel sad or empty; E15 = Nothing is much fun anymore; E16 = I have trouble sleeping; E17 = I have problems with my appetite; E18 = I have no energy for things; E19 = I am tyred a lot; E20 = I cannot think clearly; E21 = I feel worthless; E22 = I feel like I don't want to move; E23 = I feel restless. *Behavioural problems (Strength and Difficulties Questionnaire (SDQ))*: B1 = Restless, overactive; B2 = Constantly fidgeting; B3 = Easily distracted; B4 = Thinks things out before acting (reverse coded); B5 = Good attention span, see chores or homework to the end (reverse coded); B6 = Often loses temper; B7 = Generally well behaved, usually does what adults request (reverse coded), B8 = Often fights; B9 = Often lies or cheats (argumentative); B10 = Steals from home (spiteful).

These included symptoms of hyperactivity, inattention, and conduct problems, and were measured using the teacher report version of the Strength and Difficulties Questionnaire (SDQ; Goodman, 1997). The SDQ includes 25 items, with each item rated as ‘not true’, ‘somewhat true’ or ‘certainly true’ and scored as 0, 1 or 2 respectively (Goodman, 1997). Five items belong to the hyperactivity/inattention subscale and five to conduct problems (Figure 2). In the current sample, Cronbach's alpha of the hyperactivity/inattention subscale was *α* = 0.87 and *α* = 0.74 for conduct problems. The SDQ (teacher and parent reports) is a widely used and well‐validated for ADHD symptoms (Goodman, 2000) as well as for conduct problems (Goodman et al., 2010; Mason et al., 2012).

#### Emotional symptoms

Symptoms of anxiety and depression were measured using three subscales of the Revised Child Depression and Anxiety Scale (RCADS, Chorpita et al., 2000): the Major Depressive Disorder symptoms subscale (10 items, *α* = 0.81), the Separation Anxiety Disorder (SAD) symptoms subscale (7‐item, *α* = 0.73), and the 6‐item Generalized Anxiety Disorder symptoms subscale (6‐item, *α* = 0.84) (See Figure 2 for items). We focussed on these specific subscales as prior research has shown that these symptoms are more consistently associated with sleep problems in childhood (Gregory & O’Connor, 2002). Children rated how often each item applies to them on a 4‐point Likert scale, ranging from 0 (‘Never’), 1 (‘Sometimes’), 2 (‘Often’) and 3 (‘Always’). Raw scores were converted into T‐scores (0–100), with higher scores indicating greater symptom severity. RCADS has strong structural, convergent, and discriminant validity (Chorpita et al., 2000, 2005, 2021; Kosters et al., 2015; Piqueras et al., 2017), aligning with symptoms described in the DSM‐IV (American Psychiatric Association, 2000).

### Statistical analysis

All analyses were pre‐registered https://osf.io/s5ypg. Analyses were conducted in *R* studio (Version 2024.12.0 + 467) and missing data were handled using the mice package by generating five imputed datasets through Predictive Mean Matching (Buuren & Groothuis‐Oudshoorn, 2011; Rodriguez et al., 2022).

After performing descriptive analysis, we computed our network including sleep problems, teacher‐reported externalizing symptoms (hyperactivity/inattention and conduct problems) and self‐reported internalizing symptoms (anxiety and depression) using the bootnet and qgraph packages designed by Epskamp et al. (2018). In this approach, individual symptoms or behaviours are represented as nodes, and their unique associations (regularized partial correlations) are depicted as edges of the network. An edge is a connection between two nodes, indicating a statistically meaningful relationship, while its weight reflects the strength and direction of that relationship. Partial correlations between pairs of variables indicate their unique relationship while controlling for all others in the network. For interpretability and to reduce the likelihood of detecting spurious associations, we used Gaussian Graphical Model employing the graphical least absolute shrinkage and selection operator (GLASSO) regularisation (Tibshirani, 1996), based on the Extended Bayesian Information Criterion (Chen & Chen, 2008), and ensuring the model parsimony and fit. Using LASSO regularization (via EBICglasso) ensured that weaker, potentially spurious connections are minimized, resulting in a sparse and interpretable network structure (Epskamp et al., 2016; Friedman et al., 2007). To account for non‐normally distributed data, we used Spearman correlations via cor_auto, which automatically selects an appropriate method based on the scale and distribution of the data (e.g., skewed continuous), as recommended by Epskamp et al. (2018).

We used the Louvain method by using modularity to detect clustering within the networks and identify highly interconnected nodes (Blondel et al., 2024). To assess the relationships between individual symptoms and behaviours and the importance of individual nodes within the network, we estimated centrality indices including strength, closeness, and betweenness, using the centralityPlot function in the qgraph package (Robinaugh et al., 2016). These values were z‐standardized to facilitate interpretation and comparison across nodes. Node strength refers to how strongly and frequently a node is directly connected (via edges) to other nodes. Expected influence extends this by also accounting for the direction (positive or negative) of connections, with higher values indicating nodes with many strong positive associations and lower values indicating more negative associations. Closeness represents how close a node is to all others in the network and betweenness measures how often a node lies on the shortest path between others, serving as a bridge for interaction; however, these latter metrics were not interpreted due to their low stability. We also used bridge centrality to identify nodes linking their cluster with the other clusters in the network (Hevey, 2018).

We used nonparametric bootstrapping to assess the accuracy of edge weights, generating 95% confidence intervals around each estimate, and conducted 1000 bootstrapped difference tests to statistically compare differences in edge weights and centrality indices across the network. This procedure enabled us to determine which associations and nodes were significantly stronger or weaker, thereby enhancing the robustness and interpretability of the network. To evaluate the stability of centrality estimates, we applied case‐dropping subset bootstrapping, which involved repeatedly resampling subsets of the data and recalculating centrality metrics. From these iterations, we computed the correlation stability (CS) coefficient. Following the guidelines by Epskamp et al. (2018), we considered a CS coefficient above 0.50 as the threshold for good stability and accuracy.

To evaluate the robustness of the network structure, we conducted an exploratory–confirmatory split‐sample validation procedure (Golino et al., 2020) by estimating the network separately in two randomly generated halves of the dataset to assess the stability of the findings across these two internal samples. To evaluate the robustness of how we handled missing data, we also compared networks estimated from the imputed datasets with a complete‐case network using the Network Comparison Test (NCT) (with 1000 iterations and adjusted for multiple comparisons).

Finally, we used the NCT to compare network models estimated separately for boys and girls. The test assessed two aspects: (1) global strength (S), the sum of all absolute edge weights in each network, and 2) network structure (M), the pattern and distribution of edge weights. Again, we used the NCT's built‐in permutation procedures with 1000 iterations adjusted for multiple comparisons.

## RESULTS

### Descriptive statistics

#### Behavioural and emotional symptoms

Descriptive statistics for behavioural and emotional symptoms are presented in Table 1. In terms of male‐female differences, teachers reported boys having more hyperactivity/inattention symptoms (*d* = 0.54, 95% CI [0.39, 0.70], *p* < 0.001) and conduct problems than girls (*d* = 0.26, 95% CI [0.10, 0.41], *p* < 0.001). Boys were also more likely to report higher scores on the generalized anxiety (*d* = 0.16, 95% CI [0.10, 0.30], *p* < 0.5) and separation anxiety subscales (*d* = 0.24, 95% CI [0.10, 0.39], *p* < 0.001), with no significant sex differences in depression symptoms (*d* = −0.02, 95% CI [–0.17, 0.13], *p* = 0.7). Correlations with age suggested that younger children tended to report higher generalised anxiety scores (*r* = −0.08, *p* = 0.03), but fewer conduct problems (*r* = 0.08, *p* = 0.04). Free school meal status, a proxy for lower socioeconomic status was not significantly linked to any of the variables of interest (all *p* > 0.06), with effect sizes ranging from *d* = −0.17 to 0.11 (95% CIs from −0.34 to 0.28). There was a statistically significant effect of ethnicity on hyperactivity/inattention scores, *F* (3, 705) = 6.29, *p* < 0.001, *η* ^2^ = 0.03, 95% CI [0.01, 1.00]. Post hoc Tukey tests showed that children Black/African/Caribbean/Black British (*M* = 3.61 (3.28)) and White (*M* = 3.10 (3.08)) backgrounds scored significantly higher than those from Asian/Asian British backgrounds (*M* = 2.31 (2.56)), who comprised the largest group in the sample. No other group differences reached significance (all *p* > 0.31).

**TABLE 1:** Descriptive statistics (*N* = 876).

Variable	*N*	Range	Mean (SD)		
			Total	Male	Female
Child‐reported					
Total sleep problems (items 4–23)	765	23–57	36.54 (6.91)	35.73 (6.67)	37.25 (7.16)
Generalized anxiety	768	28.23–83.74	43.67 (10.79)	44.50 (11.52)	42.75 (10.08)
Separation anxiety	768	33.64–95.25	49.98 (9.88)	50.25 (10.00)	47.83 (9.59)
Depression	768	29.83–93.76	48.66 (11.62)	48.52 (12.17)	48.76 (11.03)
Teacher‐reported					
Hyperactivity/inattention	712	0–10	2.61 (2.74)	3.35 (2.95)	1.90 (2.31)
Conduct problems	712	0–9	0.80 (1.47)	1.00 (1.56)	0.62 (1.35)

Abbreviation: SD, Standard Deviation.

#### Sleep problems

The average scores for individual sleep problem items from the child‐reported SSR that were included in the network analysis are presented in Figure 1. In addition to the 23 items of the SSR that describe specific sleep problems, three additional questions give context to children's responses. These indicated that 76% of children reported they ‘liked going to sleep’, and 70% indicated they had no trouble sleeping. Most children identified their mother as the primary decision‐maker (62.8%), followed by their father (16.7%) and themselves (7.6%). There were only sex and age effects in relation to total sleep scores: girls reported having more sleep problems than boys (*d* = −0.22, 95% CI [−0.36, −0.07], *p* < 0.01) and sleep problem scores correlated negatively with age (*r* = −0.17, *p* < 0.001).

### Main research questions: Network measures of clusters and centrality

The estimated network (Figure 2) included 56 nodes and 539 non‐zero edges. The network included edges with a mean weight of 0.04, indicating that, on average, associations between connected nodes were modest; these edges represent regularized partial correlations rather than raw correlations, capturing direct relationships after controlling for all other variables.

#### Structure and symptom clusters

The network revealed seven distinct symptom clusters, some corresponding clearly to conceptual domains. There were three clusters (5, 6 and 7) that captured emotional symptoms from the Revised Children's Anxiety and Depression Subscales (RCADs, *Clusters 5 and 6*) and behavioural symptoms from the hyperactivity/inattention and conduct problems subscales of the SDQ (SDQ, *Cluster 7*). Of note, *Cluster 5* primarily comprised items reflecting affective (low mood) and physical (appetite, energy and daytime sleepiness, the latter from the SSR), whereas *Cluster 6* seemed to capture cognitive aspects (worries, negative cognitions) of anxiety and mood problems. Sleep items were distributed across the 4 remaining clusters. *Cluster 1* was perhaps clearest, containing items from the SSR pertaining mainly to bedtime resistance; in fact, 3 of the 4 items came from the SSR bedtime resistance subscale with the remaining item from the daytime sleepiness subscale. *Cluster 2* was dominated by sleep anxiety and night‐waking items, but interestingly, also contained separation anxiety symptoms (RCADS items 7–9, 12–13). *Cluster 3* included a mixture of sleep items from multiple SSR domains (bedtime resistance, sleep onset delay, sleep duration, night waking, other sleep behaviour, and daytime sleepiness) alongside a depression item relating to trouble sleeping (RCADS item 16). Finally, *Cluster 4* comprised three sleep items across three sleep domains: bedtime resistance, sleep duration, and daytime sleepiness.

In summary, the emotional and behavioural symptoms clustered largely consistently with our hypothesised domains. Although sleep problems were more widely dispersed throughout the network, certain clusters comprised both sleep problems and emotional symptom nodes, showing patterns of co‐occurrence between particular sleep behaviours and emotional symptoms (e.g., Cluster 2).

#### Stability indices

**FIGURE 3:**
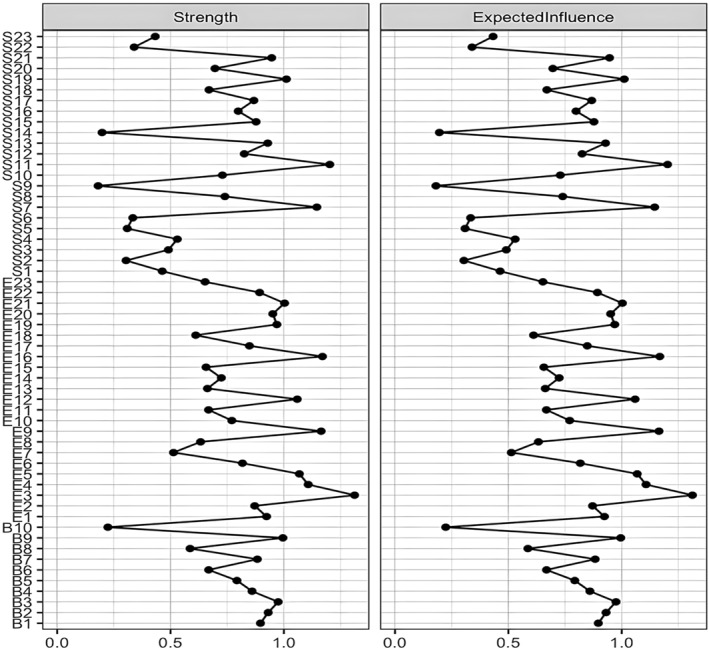
Centrality indices. Seven cluster, *noted S* for individual sleep problems (SSR), E for emotional symptoms (RCADS), and B for behavioural symptoms (SDQs).

The CS coefficients indicated good stability of the network for strength (CS = 0.60) and expected influence (CS = 0.61), suggesting these metrics were sufficiently robust. However, for closeness and betweenness, the values were below the recommended threshold of 0.5 and therefore will not be interpreted further though for transparency these are included in (Figure S1). The node‐level centrality indices that identify items most connected to others in the network, are presented in Figure 3. The topmost central items based on node strength but also expected influence were: ‘*Worry that bad things will happen to me’*, a generalised anxiety item from the RCADS, (*strength* = 1.31), ‘*Afraid of sleeping alone*’ from the sleep anxiety subscale (*strength* = 1.20), ‘*Trouble sleeping*’ from the depression subscale of the RCADS (*strength* = 1.17), and ‘*Scared if I have to sleep on my own*’, an item about separation anxiety from the RCADS (*strength* = 1.16). It is worth noting that strength measures the magnitude of an item's direct connections, while expected influence accounts for both magnitude and direction, including indirect effects. Because all associations in our network were in the same direction, strength and expected influence values were nearly identical, indicating that directionality did not provide additional insight beyond the magnitude of direct connections ((Robinaugh et al., 2016).

#### Bridge nodes

Exploratory analyses examined the main bridge nodes, identified as linking their community with other communities in the network (Figure S2). Specifically, ‘Problems with appetite’, a depression item from the RCADS (bridge strength = 10.09), ‘Sleeping during day’ a daytime sleepiness item from the SSR (bridge strength = 9.17), ‘Having trouble sleeping’, also a depression item from the RCADS (bridge strength = 9.62) and ‘Worry to go to bed at night’, a separation anxiety item from RCADS (bridge strength = 9.15) emerged as potential key bridge nodes. However, interpretation of these results should be treated as preliminary. The centrality metrics commonly used to identify bridge nodes, such as betweenness and closeness were below the recommended threshold (CS < 0.50), and therefore these findings require validation in larger samples or using alternative regularization approaches.

### Sensitivity analysis

The NCT from our exploratory–confirmatory split‐sample validation showed no differences in global strength or structure (*S* = 0; *M* = 0; both *p* = 1), indicating that key edges and central nodes, as well as the composition/nature of the networks, were stable across samples (Appendix S1, Figures S3 and S4). Similarly, as missing data ranged from 12% to 19% across sleep, emotional, and behavioural items, we compared networks estimated from imputed versus complete‐case data, which also showed no differences in global strength (*S* = 0, *p* = 1) or network structure (*M* = 0, *p* = 1), indicating robustness (Appendix S2).

### Exploratory research questions: Network comparison between boys and girls

We fitted two separate network models for boys (*n* = 441) and girls (*n* = 435). Using the NCT, overall connectivity (sum of absolute edge weights) was not statistically significant (*S* = 7.52, *p* = .30). Although overall network connectivity was not significantly different between boys and girls, examination of node centrality revealed some descriptive differences. For boys, the most central nodes were ‘worrying bad things happen’, ‘worrying about what is going to happen’, ‘worrying that something bad will happen’, ‘distracted’, and ‘afraid of sleeping alone’. For girls, the top nodes were ‘worrying bad things happen’, ‘hard to go to bed’, ‘afraid of sleeping alone’, ‘trouble sleeping’, and ‘worry to go to bed at night’ (Appendix S3, Table S1 and Figures S5 and S6).

## DISCUSSION

Sleep problems are widely recognised as a transdiagnostic feature of childhood behavioural and emotional symptoms. As existing studies have measured sleep problems using a composite score and/or have included behavioural and emotional symptoms as individual outcomes without accounting for their co‐occurrence, evidence supporting transdiagnostic claims in relation to childhood psychopathology is limited. We used a data‐driven network approach to map relationships between individual sleep problems and individual emotional and behavioural symptoms in children. Centrality indices of strength and expected influence suggested that, alongside ‘worrying that bad things will happen to me’, other specific sleep problems played a key role in other aspects of sleep disturbance and emotional and behavioural symptoms. These related to sleeping alone (‘afraid of sleeping alone’, ‘scared if I have to sleep alone’) and perceived difficulties sleeping (‘having trouble sleeping’). Exploratory bridge‐node analyses suggested that ‘having trouble sleeping’, ‘feeling sleepy during day’ and ‘worrying when going to bed at night’ were key items linking different communities, in addition to problems with appetite may act as potential items linking different communities within the network. However, these findings should be interpreted cautiously, as the centrality metrics used to identify bridge nodes showed limited stability, and the results remain preliminary. In terms of clustering, we identified four clusters of sleep problems and a cohesive, separate cluster of behavioural symptoms. Emotional symptoms clustered into affective and physical aspects (low mood, loss of appetite, fatigue) and cognitive aspects (worry, negative cognitions). Two sleep clusters emerged with clearer themes of bedtime resistance and sleep anxiety, while two contained mixed items. We found similarity between boys and girls in observed symptom interrelations. Even though sex differences were not statistically significant, boys showed slightly higher centrality for worry‐related items. A behavioural symptom also appeared among their top nodes, consistent with evidence that boys typically display higher levels of externalising behaviours during childhood (Zahn‐Waxler et al., 2008). In contrast, girls' most central nodes included more sleep‐related items, such as trouble sleeping and bedtime anxiety, aligning with research showing that girls experience greater sleep disruption and anxiety symptoms during school‐age years (Alfano et al., 2010), potentially influenced by emerging pubertal hormonal changes affecting sleep regulation and emotional reactivity (Colrain & Baker, 2011). However, these findings should be interpreted cautiously and considered exploratory.

Our findings align with the recognised developmental distinctions between emotional and behavioural problems despite their comorbidity (Achenbach et al., 2016; Gillberg et al., 2004). What is novel, however, about our findings is how each set of these internalising and externalising symptoms relates to specific sleep problems. Other studies have focussed on associations between sleep and psychopathology symptoms in clinical neurodiverse samples (autism) and have also highlighted strong links between sleep problems and emotional symptoms (Richdale et al., 2024; Sommers et al., 2025). Here, we extended this work by examining item‐level associations in a non‐clinical unselected sample. Our network analysis suggests that emotional symptoms were more proximally interwoven with various types of sleep problems across multiple clusters. By contrast, behavioural symptoms formed a dense but relatively isolated and peripheral cluster, with weaker links to sleep problems (i.e., bedtime resistance, daytime tiredness). These findings are somewhat unexpected, given earlier findings that these symptoms are associated with shorter sleep duration (Sidol et al., 2023; Touchette et al., 2007), parasomnias and night wakings (Smedje et al., 2001), bedtime resistance (Dong et al., 2024; Fletcher et al., 2018), sleep anxiety (Wagner & Schlarb, 2012), and daytime sleepiness (Becker et al., 2020; Dong et al., 2024). It is possible that earlier studies did not account for confounding effects of all individual sleep problems, or co‐occurring anxiety and depression symptoms. That emotional symptoms especially symptoms relating to separation anxiety and depression are more tightly interwoven with sleep difficulties than behavioural issues (hyperactivity/inattention and conduct problems) are consistent with DSM diagnostic criteria (APA, 2022), but also behavioural genetics findings (Madrid‐Valero, 2019). Genetic correlations between sleep disturbances and emotional problems have been found to be stronger than those between sleep and behavioural problems. This suggests a closer shared genetic liability with internalising symptoms (Gregory et al., 2009; Lind et al., 2017). Sleep problems may be part of the same phenotypic and genetic cluster of internalising symptoms.

Amid a closer‐knit relationship between emotional symptoms and sleep problems, some specific sleep problems were particularly inter‐connected and bridged different communities of items. Sleep anxiety emerged as a central node within the symptom network. Crowe and Spiro‐Levitt (2024) demonstrated that children with SAD exhibit significantly higher rates of sleep problems including bedtime resistance, nightmares, and parasomnias, driven by fears of separation, hyperarousal, and cognitive rumination at night. These disturbances not only reduce sleep quality and restorative function but also predict future emotional symptoms, such as generalized anxiety and mood disorders (Leahy & Gradisar, 2012; Shanahan et al., 2014). While sleep anxiety appears a central feature in the network, other symptoms such as problems with appetite, trouble sleeping, daytime sleepiness, and separation anxiety about going to bed at night are also strongly connected across other sleep items and psychopathology. These patterns suggest that certain specific sleep behaviours and appetite may serve as shared points of vulnerability linking emotional and behavioural difficulties to differing degrees. This makes them important targets for early intervention in school‐aged children.

There are some key limitations of this study. First, the cross‐sectional design used limits any conclusions regarding temporality between emotional and behavioural symptoms and sleep problems. Previous studies have found that sleep problems can be concurrent, consequential but also precursory to childhood psychopathology, such that they can maintain and exacerbate symptoms as well as their impact on functioning (Blacher et al., 2024; Lam & Lam, 2021). It would be interesting to investigate if the specific sleep problems that are central to emotional and behavioural symptoms are also ones that maintain these associations across time. Second, we used different informant ratings for emotional versus behavioural symptoms. Self‐report is generally considered more reliable for internalizing symptoms in children, whereas teacher‐report tends to be more accurate for externalizing (behavioural) symptoms (Rhee et al., 2007; Saez et al., 2019). However, one could argue that because sleep problems were also measured through self‐report, this may have inflated the correlations between sleep problems and emotional symptoms, leaving behavioural symptoms as a more peripheral cluster in the network. Future studies should use multi‐informant approaches, including parental sleep reports, or employ multi‐method assessments (e.g., objective sleep tracking) to reduce potential rater bias and shared method variance that may confound associations between sleep, emotional, and behavioural symptoms. Replication analyses using teacher reports of emotional symptoms and self‐reports of behavioural symptoms, alongside parent reports of sleep problems, could help disentangle rater‐linked shared variance. Such analyses may also provide more actionable recommendations for children with sleep, emotional, and behavioural difficulties.

Our results were derived from a nonclinical, school‐based population where sleep problems and emotional and behavioural symptoms may be lower than clinical groups. To ensure that participants were able to self‐report on our key measures, we also excluded participants with Intellectual Disability Disorder, thus partially limiting generalisability of findings to population sub‐groups, including neurodiverse children (e.g., children with a diagnosis of autism, ADHD and other comorbidities). Nonetheless, a major strength lies in the use of a sample that has experienced many social determinants of health including poverty. Finally, it is important to note that the designation of sleep anxiety as transdiagnostic reflects patterns of shared symptom‐level associations across emotional and behavioural domains within our network framework, rather than causal pathways or equivalence between diagnostic categories.

## CONCLUSION

Here, we applied a network framework to examine relationships between sleep and childhood psychopathology in a non‐clinical unselected sample, focussing on item‐level associations. Our findings have revealed a more detailed understanding of how sleep problems, nighttime anxiety and moods are linked with mental health outcomes in school‐aged children. The symptom level approach highlights that specific sleep problems relating to sleep anxiety could function as central, transdiagnostic factor particularly in relation to emotional symptoms, consistent with Harvey (2001). Due to the cross‐sectional design, it is impossible to determine whether sleep anxiety is a causal mechanism; longitudinal studies are needed to validate whether it predicts symptom onset or maintenance. If replicated in longitudinal network analysis, our findings may support a modularised intervention approach, whereby treatment components are flexibly combined to target central symptoms, such as bedtime anxiety, allowing interventions to be tailored to individual profiles and thereby enhancing their precision and impact across emotional and behavioural symptomatology. Indeed, there are effective interventions for anxiety‐related sleep problems that focus on reinforcing healthy sleep hygiene such as appropriate bedtimes, screen‐free or quiet sleep environments tailored to developmental needs and family resources (Cartwright et al., 2025; Romanello et al., 2022). Cognitive behavioural therapy adapted for sleep and anxiety have also shown therapeutic benefits (Blake et al., 2018) as have behavioural sleep interventions (Hornsey et al., 2025).

## AUTHOR CONTRIBUTIONS

**Alina A. Marinca**: Writing—original draft. **Julia E. Michalek**: Writing—review and editing. **Alice M. Gregory**: Writing—review and editing. **Afia Ali**: Writing—review and editing; supervision. **Jennifer Y. F. Lau**: Supervision; writing—review and editing.

## CONFLICT OF INTEREST STATEMENT

A.G. is an advisor for a project initially sponsored by Johnson's Baby. She was a consultant for Perrigo (2021+). She receives royalties for two books Nodding Off (Bloomsbury Sigma, 2018) and The Sleepy Pebble (Flying Eye, 2019) and a sleep gift (The Gift of Sleep, Lawrence King Publishers, 2023). She was previously a CEO of Sleep Universal LTD (2022). She is a regular contributor to BBC Focus Magazine and has contributed to other outlets (such as The Conversation, The Guardian, Balance Magazine, Psyche Aeon). She occasionally receives sample products related to sleep (e.g., blue light blocking glasses) and has given a paid talk to a business (Investec). She has contributed a paid article to Neurodiem. The remaining authors have declared that they have no competing or potential conflicts of interest.

## ETHICAL CONSIDERATIONS

1. Informed consent/assent: Parental consent and child assent were obtained before participation. Participants were recruited from 10 schools across East London using a combination of opt‐in and opt‐out consent procedures.
2. Ethical approval body: QMREC, Queen Mary University of London, UK.
3. Date of Approval: June 27, 2022.
4. Approval/Reference Number: QMERC22.251.

## TRIAL REGISTRATION

1. Registry Name: OSF.
2. Registration/Identifier Number: s5ypg.
3. Date of Registration: Apr 13, 2025.
4. Link: https://osf.io/s5ypg.

## Supporting information

Supporting Information S1

## Data Availability

The data that support the findings of this study are available from the corresponding author upon reasonable request.
