# Supplementary material for: Sleep disturbance as a transdiagnostic marker of children's mental health difficulties: A network analysis of item‐level associations between different types of sleep problems and different behavioural and emotional symptoms
Source: JCPP Adv. 2026 Mar 4:e70104. Online ahead of print. doi: 10.1002/jcv2.70104 (PMC13339339; doi:10.1002/jcv2.70104)

**Sleep disturbance as a transdiagnostic marker of children’s mental health difficulties: A network analysis of item-level associations between different types of sleep problems and different behavioural and emotional symptoms**

**Supporting Information**

**Figure S1. Centrality Indices for Network Nodes**


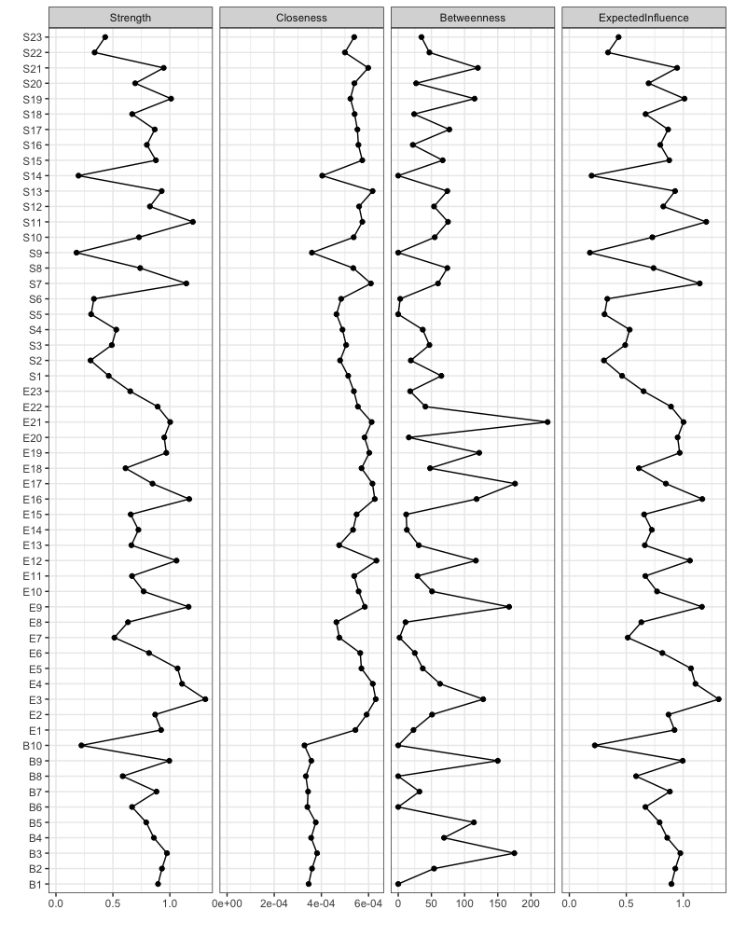


Note: Strength, closeness, betweenness, and expected influence were calculated; Centrality strength is presented due to its stability, *noted S for individual sleep problems (SSR), E for emotional symptoms (RCADS), and B for behavioural symptoms (SDQs).*

**Figure S2. Bridge Centrality Indices for Network Nodes**


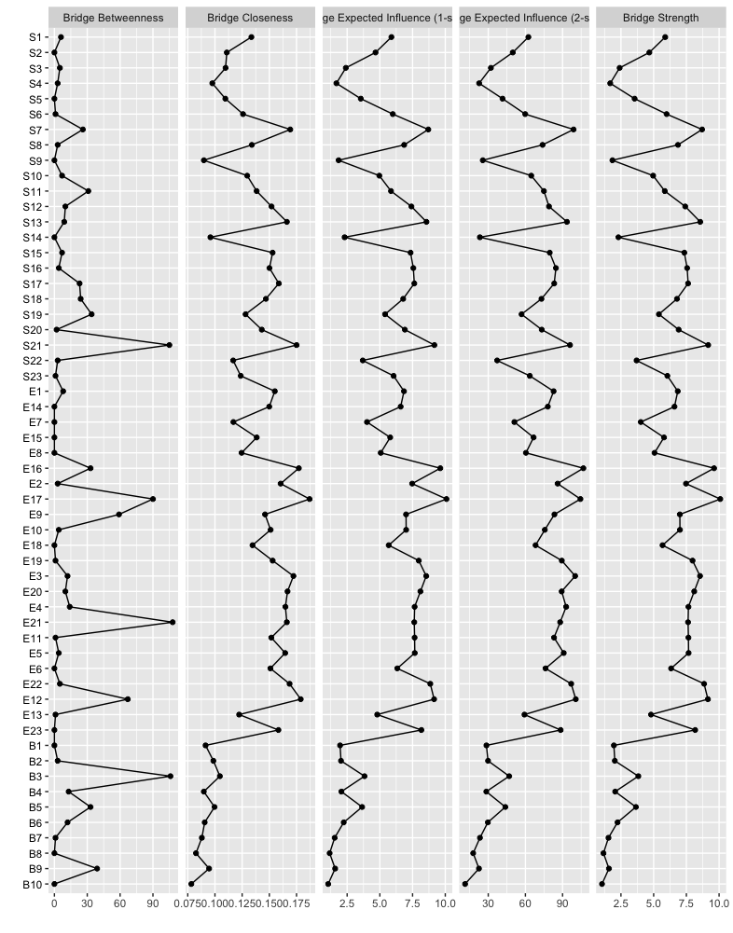


Note: Bridge betweenness, bridge closeness, bridge expected influence, and bridge strength were calculated; Bridge strength is reported, *noted S for individual sleep problems (SSR), E for emotional symptoms (RCADS), and B for behavioural symptoms (SDQs)*

**Appendix S1. Sensitivity analysis: Split-Sample Exploratory–Confirmatory Validation**

To test the robustness and replicability of the network results, we conducted an exploratory–confirmatory split-sample sensitivity analysis (Golino et al., 2020). The full sample (N = 876) was randomly divided into two equal halves using a fixed seed, resulting in an exploratory sample (n = 438) and a confirmatory sample (n = 438). Networks were estimated separately for each subset using the same EBICglasso procedure with Spearman correlations across all 56 items. The networks were formally compared using the Network Comparison Test (NCT) with 1000 bootstrap iterations to evaluate both overall network structure and global strength. The NCT indicated no significant differences between the two halves in either structure invariance (M = 0, p = 1) or global strength invariance (S = 0, p = 1). The exploratory and confirmatory split-sample networks are presented in Figures S3 and S4.

**Figure S3. Exploratory split-sample network (random half 1)**


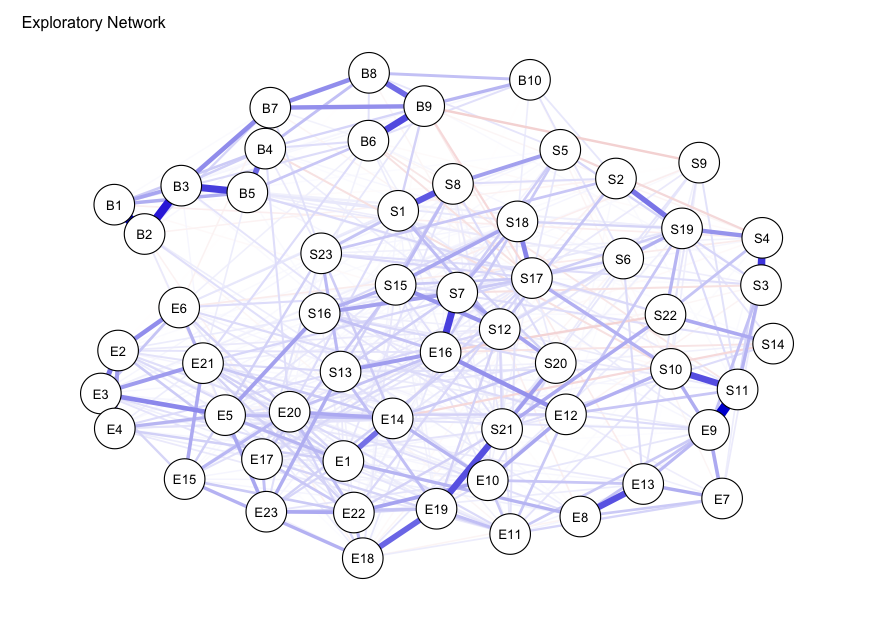


**Figure S4. Confirmatory split-sample network (random half 2).**


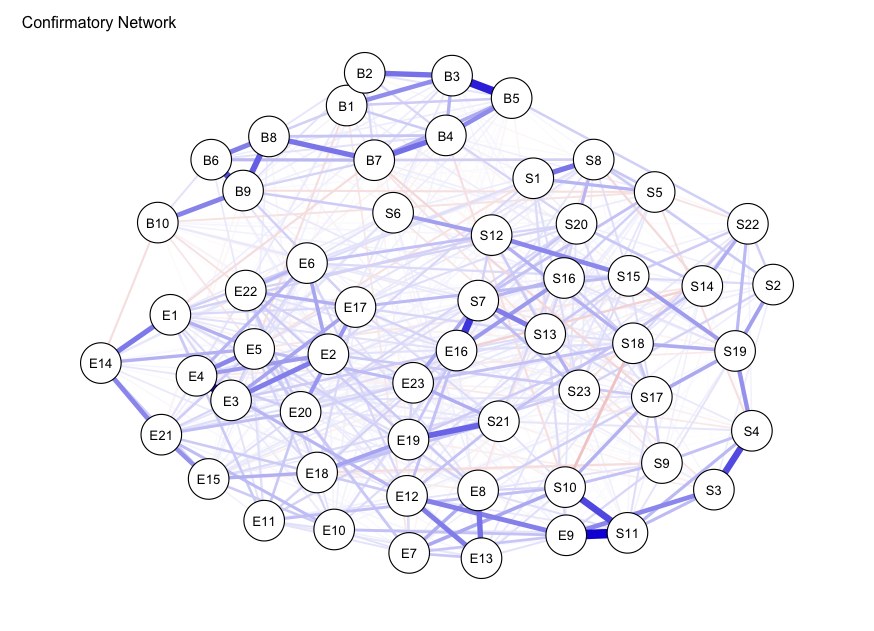


**Appendix S2. Sensitivity analysis:** Imputation - Complete-Case Network Comparison

Missing data ranged from 12–19% across sleep, emotional, and behavioural items. To assess the robustness of the network to missing data handling, we compared a network estimated from multiply imputed data (m = 5 imputations using predictive mean matching, seed = 123) with a network estimated from complete-case data. We used EBICglasso procedure with Spearman correlations across all 56 items to estimate the networks. The Network Comparison Test (NCT) with 1000 bootstrap iterations indicated no significant differences between the imputed and complete-case networks. The structure invariance test yielded (M = 0, p = 1), and the global strength invariance test yielded (S = 0, p = 1), indicating that the networks did not differ significantly indicating the robustness of the main network to missing-data handling.

**Appendix S3. Network comparison between boys and girls**

The overall connectivity (sum of absolute edge weights was not statistically significant. (S=7.52, p=.3). Networks were estimated separately for boys (n = 441) and girls (n = 435).

**Table S1**. **Top 5 most central nodes (Strength) for boys and girls**

| **Node Label (Boys)** | **Strength (Boys)** | **Girls (Node Label)** | **Strength (Girls)** |
| --- | --- | --- | --- |
| worrying bad things happen | 1.289 | worrying bad things happen | 1.280 |
| worrying about what is going to happen | 1.076 | hard to go to bed | 1.221 |
| worrying that something bad will happen | 0.976 | afraid of sleeping alone | 1.132 |
| distracted | 0.959 | trouble sleeping | 1.187 |
| afraid of sleeping alone | 0.900 | worry to go to bed at night | 1.126 |

**Figure S5. Network visualizations for boys**


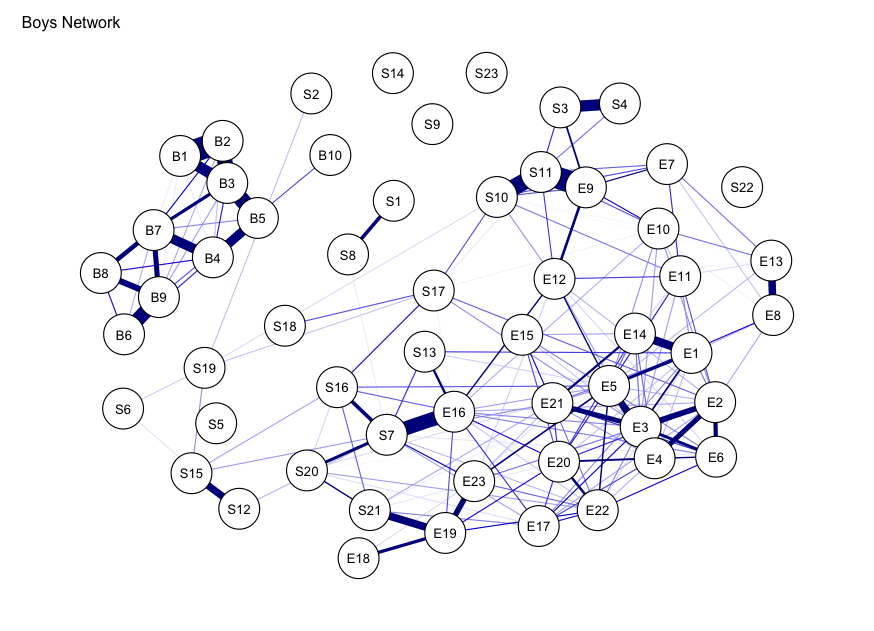


**Figure S6. Network visualizations for girls**


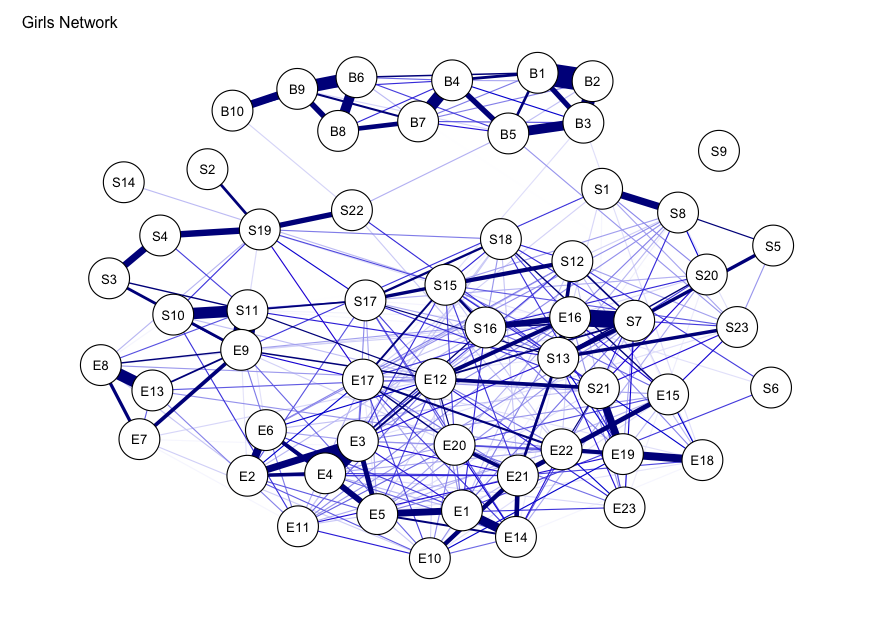

Supplement: Supplementary file 1 — Supporting Information S1 [file JCV2-9999-e70104-s001.docx]
